# Role of Virtual Reality in Treating Anxiety in Child & Adolescent

**DOI:** 10.1192/j.eurpsy.2024.877

**Published:** 2024-08-27

**Authors:** K. Shah, P. Reddy, A. Giri, S. Srinivas

**Affiliations:** ^1^ Wake Forest University; ^2^ Baptist Health - UAMS Medical Education Program PGY-4 Psychiatry Resident; ^3^ Dhaka University; ^4^Baptist Health and the University of Arkansas for Medical Sciences (BH-UAMS), Winston-Salem, United States

## Abstract

**Introduction:**

Anxiety disorder affects nearly 9.4% of children aged 3-17 years.1 Virtual Reality (VR) provides an alternative for managing anxiety due to immersive, multisensory, and excellent distraction.

**Objectives:**

The aim is to evaluate the efficacy of VR therapy in managing anxiety in children.

**Methods:**

We searched PubMed, Medline, Embase, Web of Science, and Biosisdatabases with the keywords “Virtual Reality” in the context of “AnxietyDisorders” and included 8 relevant studies published in English until February 10, 2023, for our qualitative synthesis.

**Results:**

The VR-Guided relaxation (VR-GR) effectively decreased anxiety immediately after administration. In another trial, 4 of the 9 patients completely overcame their fears, and 8 of 9 saw an improvement in target behaviors in the autism population even after six weeks after the therapy, and the effect lasted 1 year post-treatment. In another study, VR-based therapy helped reduce anxiety and behavioral scores significantly in the VR group vs. the control. In another study, they found during pediatric intravenous catheter placement, patients who received VR therapy showed significantly less anxiety and pain compared to those who did not. In another study, they found VR therapy helped reduce anxiety during the induction of pre-operative anesthesia in children undergoing elective surgery.

**Conclusions:**

A study discovered benefits with statistically significant results in reducing anxiety in children immediately after VR-based therapy. To explore the full spectrum of benefits and efficacy of VR-based therapy for anxiety as a standalone or adjunct to pharmacotherapy, we recommend future trials with robust study designs.

**Disclosure of Interest:**

None Declared

